# Rhodium, iridium and nickel complexes with a 1,3,5-triphenylbenzene tris-MIC ligand. Study of the electronic properties and catalytic activities

**DOI:** 10.3762/bjoc.11.278

**Published:** 2015-12-14

**Authors:** Carmen Mejuto, Beatriz Royo, Gregorio Guisado-Barrios, Eduardo Peris

**Affiliations:** 1 Instituto de Materiales Avanzados (INAM), Universitat Jaume I, Avda. Vicente Sos Baynat, Castellón, 12071, Spain; 2 Instituto de Tecnologia Química e Biologica da Universidade Nova de Lisboa, Av. da República, EAN, Oeiras, 2780-157, Portugal

**Keywords:** arylation of unsaturated ketones, mesoionic carbenes, nickel, iridium, rhodium

## Abstract

The coordination versatility of a 1,3,5-triphenylbenzene-tris-mesoionic carbene ligand is illustrated by the preparation of complexes with three different metals: rhodium, iridium and nickel. The rhodium and iridium complexes contained the [MCl(COD)] fragments, while the nickel compound contained [NiCpCl]. The preparation of the tris-MIC (MIC = mesoionic carbene) complex with three [IrCl(CO)_2_] fragments, allowed the estimation of the Tolman electronic parameter (TEP) for the ligand, which was compared with the TEP value for a related 1,3,5-triphenylbenzene-tris-NHC ligand. The electronic properties of the tris-MIC ligand were studied by cyclic voltammetry measurements. In all cases, the tris-MIC ligand showed a stronger electron-donating character than the corresponding NHC-based ligands. The catalytic activity of the tri-rhodium complex was tested in the addition reaction of arylboronic acids to α,β-unsaturated ketones.

## Introduction

**Scheme 1:**
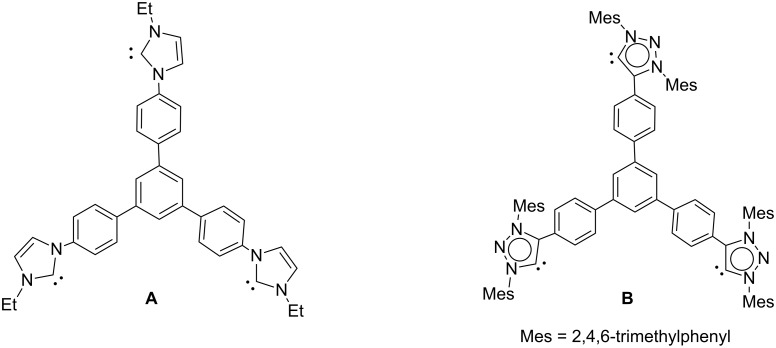
Schematic representation of ligands **A** and **B**.

Highly symmetrical poly-NHCs are a very interesting type of ligands, because they allow the preparation of a variety of supramolecular assemblies that include molecular squares and triangles [1–6], cylinder-like structures [7–13], organometallic polymers [14–22] and even organometallic mesoporous materials [21–22]. Another interesting feature of this special type of poly-NHCs is its ability to form multimetallic catalysts whose catalytic performances can be compared with analogous monometallic NHC complexes [23–25]. On several occasions their activity has proven higher than the activities shown by their monometallic counterparts [23,26]. In the last few years we became interested in the design of several types of *C*_3v_-symmetric tris-NHCs, both for the preparation of self-assembly molecular cages [12–13,27] and for the design of discrete trimetallic molecules whose catalytic performances were explored [23,25,28–29]. Among these ligands, we found those featuring a nanoscale distance between the metals especially interesting [13,27] because for these systems a catalytic cooperation between the active metal sites should not be expected. As a consequence all catalytic improvements should be assigned to reasons dealing with supramolecular interactions [30] or with the higher nanolocal concentration of metal sites in the multimetallic catalyst [31]. In this context, we obtained the 1,3,5-triphenylbenzene-based *C*_3_-symmetrical tris-NHC ligand **A** (Scheme 1), which was coordinated to rhodium and iridium [25]. The catalytic activity of the trirhodium complex was tested in the addition reaction of arylboronic acids to 2-cyclohexen-1-one, where it showed good activity. The same ligand was also used for the preparation of nanometer-sized cylinder-like structures of Cu, Ag and Au [13].

As mesoionic carbenes (MICs) are known to be stronger electron donors compared to NHCs [32–36], and because poly-MIC ligands are less explored [37–46], we very recently synthesized the tris-diarylated-(1,2,3-triazol-5-ylidene)-1,3,5-triphenylbenzene-based ligand (**B**). This ligand afforded trisilver and trigold cages with very interesting rearranging properties when mixed with the related 1,3,5-triphenylbenzene-based tris-NHC Ag cages [27]. Shortly afterwards, Sarkar and co-workers used a dialkylated (1,2,3-triazol-5-ylidene)-1,3,5-triphenylbenzene-based ligand for the preparation of the corresponding tris-Ir(III) and Pd(II) complexes and tested them for their catalytic activities [47].

Based on these previous findings, we herein report the synthesis of the tri-metallic complexes of Rh(I), Ir(I) and Ni(II) with the tris-MIC ligand **B**. The preparation of these complexes gives us an excellent opportunity to compare the electronic properties of the tris-MIC ligand **B** with those of its tris-NHC analogue, **A**. The catalytic activity of the tris-MIC-trirhodium complex was tested in the addition reaction of arylboronic acids to 2-cyclohexen-1-one and compared to the results obtained with the tris-NHC analogue.

## Results and Discussion

**Scheme 2:**
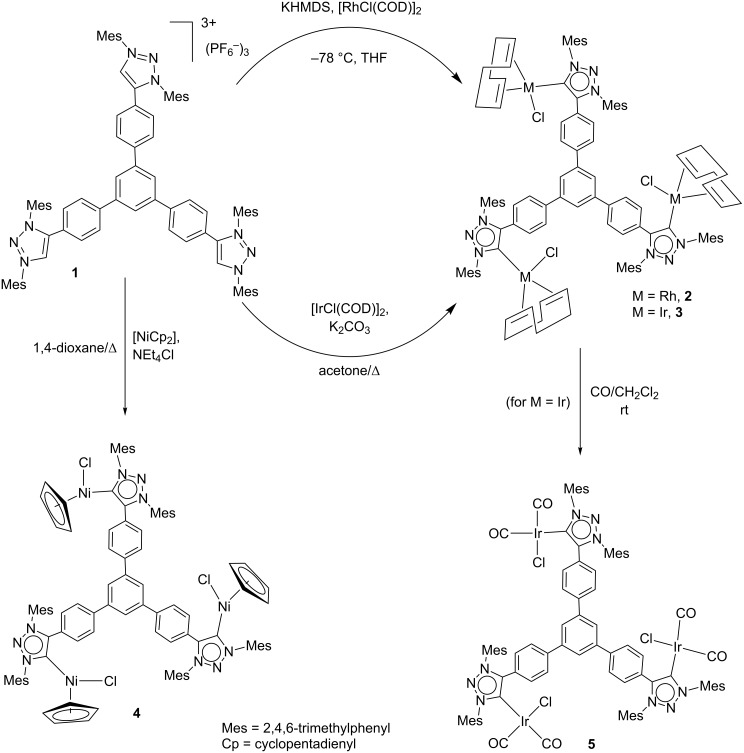
Synthesis of rhodium(I), iridium(I), and nickel(II) complexes of ligand **B**.

Complex **2** was obtained by the in situ deprotonation of the tris-triazolium salt **1** with potassium bis(trimethyl)silyl amide (KHMDS) in the presence of [RhCl(COD)]_2_ in THF at −78 °C (Scheme 2). It was isolated in 84% yield after purification by column chromatography. For the preparation of the related iridium(I) complex, we found it more convenient to use a preparative method inspired by a recent work by Plenio and co-workers [48]. In this way refluxing a mixture of **1** with [IrCl(COD)]_2_ in the presence of K_2_CO_3_ in acetone for 24 h gave the triiridium(I) complex **3** in 60% yield after purification. The complexes **2** and **3** were characterized by NMR and mass spectrometry. Both, the ^1^H and the ^13^C NMR spectra of the complexes were consistent with the expected threefold symmetry of the molecules, as exemplified by the appearance of one only signal for the carbene carbons, at 173.4 (^1^*J*_Rh–C_ = 41.5 Hz) and 172.1 ppm, for **2** and **3**, respectively. The ^1^H NMR spectra of complexes **2** and **3** exhibited relatively broad signals, which may indicate a fluxional behavior. This may be likely caused by the combined rotation around the C–C sigma bonds of the tris-MIC ligand and the C_carbene_–M bond.

In order to widen the coordination scope of the tris-MIC ligand **B**, the corresponding Ni(II) complex was also synthesized. The reaction of **1** with [NiCp_2_] in the presence of NEt_4_Cl in refluxing dioxane, afforded the tris-MIC complex of Ni(II) **4**, as a red solid in 40% yield after purification. The three-fold symmetry of this complex was also confirmed by its NMR spectra. In the 1H NMR a singlet assigned to the equivalent 15 protons of the three cyclopentadienyl rings was observed. As already indicated for complexes **2** and **3** the ^1^H NMR spectrum of complex **4** showed broad signals as a consequence of the fluxionality due to the rotation about the C–C sigma bonds of the ligand, and the C_carbene_–Ni bond. The ^13^C NMR spectrum showed the distinctive signal due to the metalation of the carbene carbon at 151.4 ppm, which is in the same region of the previously reported [NiCpCl(MIC)] complex (148 ppm) [49]. The trimetallic nature of the complex was further confirmed by mass spectrometry, which revealed a peak at *m*/*z* 810.7, assigned to [M − 2Cl]^2+^. Compound **4** is very interesting, because despite the fact that many [NiCpX(NHC)] complexes have already been reported [49–53], to our knowledge, this is the first tris-MIC-trinickel complex described so far.

**Scheme 3:**
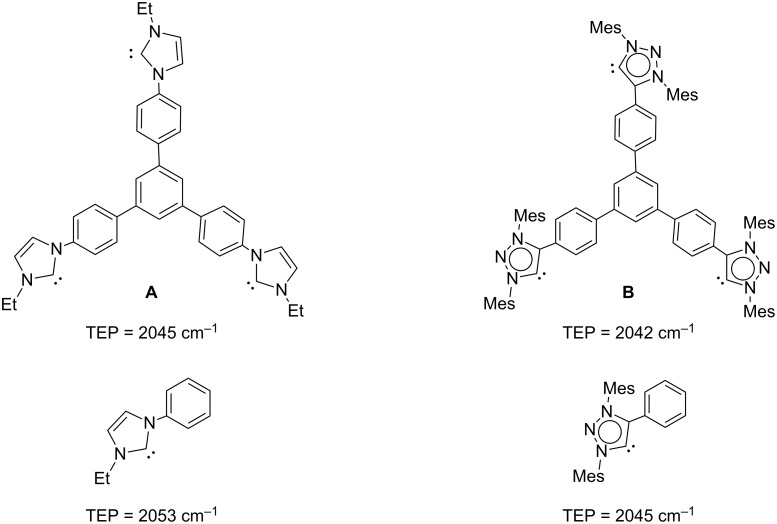
Tolman electronic parameters (TEP) for **A**, **B** and their related monocarbenes.

To further evaluate the electron-donating character of the tris-MIC ligand **B**, the iridium hexacarbonyl complex **5** was obtained by bubbling carbon monoxide into a solution of **3** in CH_2_Cl_2_. The resulting yellow solid was obtained in 93% yield. The IR spectrum of a CH_2_Cl_2_ solution of **5** showed the characteristic CO stretching bands at 2057 and 1972 cm^−1^ from which a Tolman electronic parameter (TEP) of 2042 cm^−1^ could be estimated by using the well-accepted correlations [54–56]. This obtained TEP value is slightly lower than the one shown by the tris-NHC analogue **A**, for which the reported TEP was 2045 cm^−1^, therefore suggesting that the tris-MIC ligand **B** is a stronger electron donor than ligand **A**. However, this comparison must be taken with care, because the tris-carbene ligands **A** and **B**, not only differ in the nature of their carbenes (MIC vs NHC), but also in their substituents at the carbene rings, which may also affect the electronic nature of the ligands. It worth mentioning, that the monometallic complex [IrCl(MIC)(CO)_2_] (MIC = 1,3-bis(2,6-diisopropylphenyl)-4-phenyl-1,2,3-triazolylidene), which may be considered as the monometallic analogue of complex **5**, displays an average CO stretching frequency at 2018 cm^−1^ [32]. This frequency is 4 cm^−1^ higher than the average frequency observed for **5** (2014 cm^−1^), indicating a stronger electron-donating character of the tris-MIC ligand. In a similar way, the Tolman electronic parameter of 1-ethyl-3-phenylimidazolyidene (which may be considered as the monocarbene analogue of **A**) is 2053 cm^−1^ [57], therefore 8 cm^−1^ higher than that reported for **A**. Scheme 3 displays the comparison of the TEP values of the triscarbene ligands **A** and **B**, and their related monocarbenes. The results clearly indicate that the tritopic nature of ligands **A** and **B** is significantly improving the electron-donating ability of the ligands which by no means should be regarded as the simple combination of the three monocarbenes that constitute the branches of these tris-carbenes.

**Figure 1:**
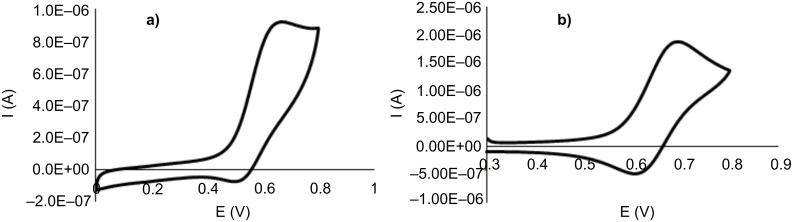
CV plots of complexes **2** (a), and **3** (b). Experiments were carried out using 1 mM solutions of the complexes in dry CH_2_Cl_2_ with 0.1 M [NBu_4_][PF_6_] as the supporting electrolyte, 100 mVs^−1^ scan rate, Fc^+^/Fc used as internal standard with *E*_1/2_ (Fc/Fc^+^) = 0.44 V vs SCE.

**Figure 2:**
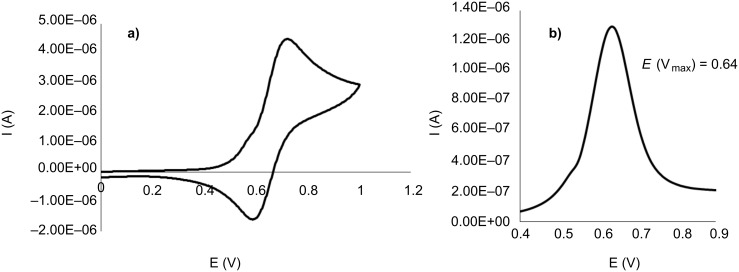
CV plot (a) and relevant DPV section (b) of complex **4**. Experiments were carried out using 1 mM solution of the complex in dry CH_2_Cl_2_ with 0.1 M [NBu_4_][PF_6_] as the supporting electrolyte, 100 mVs^−1^ scan rate, Fc^+^/ Fc used as internal standard with *E*_1/2_ (Fc/Fc^+^)= 0.44V vs SCE.

To gain further insight into the electronic properties of the tris-MIC ligand **B**, cyclic voltammetry studies of **2** and **3** (Figure 1) and **4** (Figure 2) were performed. The rhodium complex **2** showed an irreversible wave at *E*_1/2_ = 0.56 mV, while the iridium complex **3** displayed a pseudo-reversible wave at a half-wave potential of *E*_1/2_ = 0.63 mV. Compared to the cyclic voltammetry data obtained for the analogous Rh and Ir complexes with the tris-NHC ligand **A** (*E*_1/2_ = 0.67 mV, for both complexes) [25], the observed lower *E*_1/2_ values for complexes **2** and **3** are consistent with a stronger electron-donating character of the tris-MIC ligand **B**. Further the observation of only one redox wave for both complexes **2** and **3** is consistent with the electronic disconnection of the three metals in both trimetallic complexes.

The cyclic voltammetry diagram of the tri-Ni(II) complex **4** is shown in Figure 2, together with the differential pulse voltammetry (DPV) plot. The complex shows a quasi-reversible wave at a half-wave potential of *E*_1/2_ = 0.64 mV, which is significantly lower than the half-wave potential (*E*_1/2_ = 0.72 mV) exhibited by the monometallic NHC-based complex [NiCpCl(IMes)] [50] (IMes = 1,3-mesitylimidazolylidene). This is in agreement with the stronger electron-donating character for the ligand in **4** compared to IMes. From the differential pulse voltammetry (DPV) analysis generated for **4** it can be seen that there is only one redox event taking place, thus evidencing that the trimetallic complex **4** contains three nickel fragments that are essentially decoupled.

**Scheme 4:**
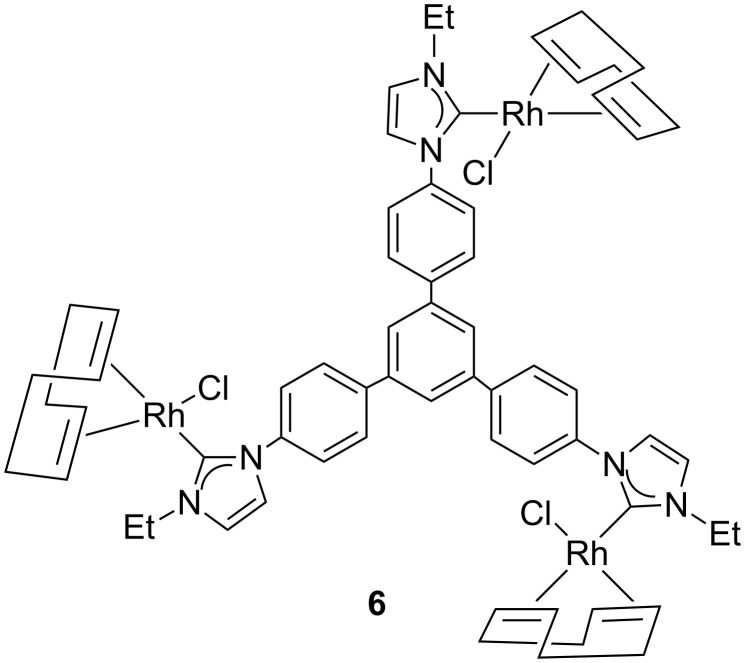
Schematic representation of complex **6**.

Since we previously evaluated the catalytic properties of the tris-NHC Rh(I) complex **6** (Scheme 4) in the rhodium-catalyzed addition of arylboronic acids to α,β-unsaturated ketones [25] we decided to study complex **2** in the same reaction. From these comparative data it was assumed to gaining more information about the effects of the nature of the carbene ligand while maintaining a similar structural environment on the catalyst. The catalytic addition of arylboronic acids to α,β-unsaturated ketones [58–62] is a process for which several Rh(I)-NHC complexes have afforded excellent activities and chemoselectivities [58,63–64].

For the catalytic experiments, the arylation of 2-cyclohexen-1-one with several arylboronic acids was studied. The results obtained with 0.066 mol % catalyst **2** were compared with those previously obtained with catalyst **6**. As can be seen from the data collected in Table 1, the activity of complex **2** is lower than that shown by complex **6**, both in terms of conversion and selectivity. For all reactions carried out in the presence of catalyst **2**, deborylation of the boronic acids took place. This side reaction explains the differences found between conversions and yields for all reactions performed. This observation is more relevant for the case of the use of 4-methoxyphenylboronic acid, for which the formation of anisole is the dominant process (Table 1, entries 5 and 6).

**Table 1:** 1,4-Addition of arylboronic acids to 2-cyclohex-1-one.^a^

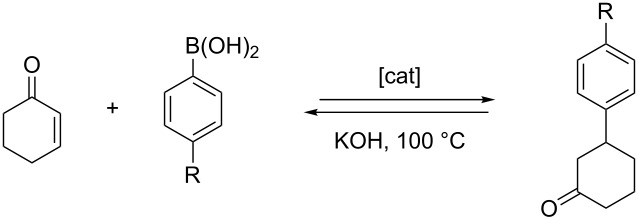			

Entry	Catalyst	R	Conversion (yield, %)

1	**6**	H	100 (91)^b^
2	**2**	H	66 (43)^c^
3	**6**	Me	85 (69)^b^
4	**2**	Me	63 (34)^c^
5	**6**	OMe	48 (16)^c^
6	**2**	OMe	52 (15)^c^

^a^Reaction conditions: Catalyst (0.066 mol %), 2-cyclohexen-1-one (0.5 mmol), KOH (0.09 mmol), ArB(OH)_2_ (0.6 mmol), dry toluene (3 mL). Conversions were determined by gas chromatography (GC), using ^b^anisol or ^c^2,4,6-trimethylphenol as internal standards. Yields are given in parentheses. The results given for the use of complex **6** were taken from reference [25].

## Conclusion

In summary, this work illustrated the high coordination versatility of a nanosized tris-MIC ligand, by obtaining a series of Rh(I), Ir(I) and Ni(II) complexes. Interestingly, the tris-MIC complex of Ni is the first trimetallic Ni complex with a tris-carbene ligand. The electron-donating properties of the ligand were assessed by cyclic voltammetry and by IR spectroscopy of the corresponding carbonylated tris-Ir(I) complex. Both techniques indicate that the ligand is a stronger electron donor than its related tris-NHC analogue. It is even more important to mention that the tris-MIC ligand is a stronger electron donor than its more closely related mono-MIC ligand – a situation that is also true for the tris-NHC ligand **A**, compared to its related mono-NHC counterpart. This indicates that the triphenylbenzene core is significantly increasing the electron-donating character of the ligand, compared to the related monocarbenes, thus proving that the tris-carbene ligands should not be regarded as a simple combination of three independent monocarbenes.

## Supporting Information

File 1 Experimental details and copies of spectra.
