# Supplementary material for: Rhodium, iridium and nickel complexes with a 1,3,5-triphenylbenzene tris-MIC ligand. Study of the electronic properties and catalytic activities
Source: Beilstein J Org Chem. 2015 Dec 14;11:2584–90. doi: 10.3762/bjoc.11.278 (PMC4685890; doi:10.3762/bjoc.11.278)
Supplement: File 1 — Experimental details and copies of spectra. [file Beilstein_J_Org_Chem-11-2584-s001.pdf]

Supporting Information

for

Rhodium, iridium and nickel complexes with a 1,3,5-triphenylbenzene tris-MIC ligand. Study of the electronic properties and catalytic activities

Carmen Mejuto[1], Beatriz Royo[2], Gregorio Guisado-Barrios[1*] and Eduardo Peris[1*]

Address: [1]Instituto de Materiales Avanzados (INAM), Universitat Jaume I, Avda. Vicente Sos Baynat, Castellón, 12071, Spain and [2]Instituto de Tecnologia Química e Biologica da Universidade Nova de Lisboa, Av. da República, EAN, Oeiras, 2780-157, Portugal

Email: Gregorio Guisado-Barrios - guisado@uji.es; Eduardo Peris - eperis@uji.es

*Corresponding author

Experimental details and copies of spectra

Table of Contents

General methods. S3
Experimental section S3
Synthesis of complex **2**. S3
Synthesis of complex **3**. S4
Synthesis of complex **4**. S4
Synthesis of complex **5**. S4
General proceduce for the addition reaction of arylboronic acids to cyclohex-2-enone. S5
References S5
Spectra S5
^{1}H and ^{13}C NMR spectra of **2** S5
^{1}H and ^{13}C NMR spectra of **3** S6
^{1}H and ^{13}C NMR spectra of **4** S7
^{1}H and ^{13}C NMR spectra of **5** S8

General methods.

All operations were carried out using standard Schlenk techniques under a nitrogen atmosphere unless otherwise stated. Solvents were dried using a solvent purification system (MBraun SPS). All reagents were used as received from commercial suppliers. NMR spectra were recorded on either a Varian Mercury 300 or Varian NMR System 500 MHz and Bruker Avance III 400 MHz spectrometers and referenced (^{1}H, ^{13}C) as follows: $CDCl_3$ (δ 7.26, 77.16). Electrospray mass spectra (ESIMS) were recorded on a Micromass Quatro LC instrument; nitrogen was employed as drying and nebulizing gas. Accurate mass measurements were performed by use of a Q-TOF premier mass spectrometer with an electrospray source (Waters, Manchester, UK) operating at a resolution of ca. 16 000 (fwhm). Elemental analyses were carried out on a EuroEA3000 Eurovector Analyzer. The tris-triazolium salt **A** was synthesized as previously reported [1].

Electrochemical studies were carried out with an Autolab Potentiostat, Model PGSTAT101 using a three-electrode cell. The cell was equipped with a platinum working and counter electrodes and a silver wire as reference electrode. In all experiments, $[NBu_4][PF_6]$ (0.1 M in dry CH_2Cl_2) was used as the supporting electrolyte with analyte concentration of approximately 1 mM. Measurements were performed between 50 to 150 mVs^{-1} scan rates. All redox potentials were referenced to the Fc^{+}/Fc couple as internal standard with $E_{1/2}(Fc/Fc^{+})$ vs. SCE = +0.44 V.

Satisfactory elemental analyses of all metal complexes were difficult to obtain due to the sensitivity of the complexes toward moisture. The elemental analyses of complexes **2**, **3** and **4** could only be adjusted after addition of several solvent molecules, which are also observed in the NMR spectra. A complete set of NMR spectra is provided.

Experimental section

Synthesis of complex 2.

To a Schlenk tube containing a solution of tris-triazolium salt **1** (0.05 g, 0.03 mmol) and $[RhCl(COD)]_2$ (0.031 g, 0.06 mmol) in THF (15 mL) at −78 °C, was added dropwise a solution of KHMDS (0.5 M in toluene, 400 µL, 0.20 mmol). The mixture was allowed to warm to room temperature and stirred overnight. After removal of the volatiles, the crude solid was dissolved in CH_2Cl_2 and purified by column chromatography using silica gel and a DCM/acetone mixture, to yield 55 mg of an orange solid. Yield: 84.4%. ^{1}H NMR (300 MHz, $CDCl_3$): δ (ppm) 8.32 (d, J = 7.9 Hz, 6H, CH_{Ar}), 7.84 (s, 3H, CH_{Ar}), 7.72 (d, J = 8.6 Hz, 6H, CH_{Ar}), 7.14-7.02 (m, 12H, CH_{Ar}), 4.82 (m, 6H, CH_{COD}), 3.27 (m, 6H, CH_{COD}), 3.39 (m, 36H, CH_3), 2.11-1.98 (m, 42H, CH_3, CH_{COD}). $^{13}C\{^{1}H\}$ NMR (75 MHz, $CDCl_3$): δ (ppm) 173.4 (d, J = 41.5 Hz, Rh- $C_{carbene}$), 145.8 (C_{Ar}), 141.6 (C_{Ar}), 141.2 (C_{Ar}), 140.0 (C_{Ar}), 136.7 (C_{Ar}), 135.0 (C_{Ar}), 134.1 (C_{Ar}), 133.9 (C_{Ar}), 132.3 (C_{Ar}), 129.9 (C_{Ar}), 128.3 (C_{Ar}), 127.9 (C_{Ar}), 126.9 (C_{Ar}), 125.3 (C_{Ar}), 95.9 (CH_{COD}), 95.4 (CH_{COD}), 69.3 (CH_{COD}), 67.1 (CH_{COD}), 33.7 (CH_{2COD}), 32.0 (CH_{2COD}), 29.4 (CH_{2COD}), 28.5 (CH_{2COD}), 21.4 (CH_3), 19.6 (CH_3), 17.9 (CH_3), 17.7 (CH_3). HRMS ESI-TOF-MS

(positive mode): 616.6 $[M]^{3+}$, 942.3 $[M-Cl]^{2+}$. Anal. Calcd. for $C_{108}H_{117}N_9Rh_3Cl_3(H_2O)(CHCl_3)_6$: C, 50.89; H, 4.68; N, 4.69. Found: C, 50.6; H, 5.1; N, 4.5.

Synthesis of complex 3.
A Schlenk tube was charged with tris-triazolium salt **1** (0.05 g, 0.03 mmol), $[IrCl(COD)]_2$ (0.043 g, 0.06 mmol) and K_2CO_3 (0.54 g, 0.39 mmol). The resulting mixture was suspended in acetone (10 mL) and stirred for 1 day at 60 °C. Afterwards the solid was filtered and washed several times with acetone. The crude mixture was dissolved in CH_2Cl_2 and purified by filtration through a pad of celite. The resulting solution was partially evaporated and hexane was added, yielding 40 mg of a yellow precipitate. Yield: 59.5%. ^{1}H NMR (300 MHz, $CDCl_3$): δ (ppm) 8.05 (d, J = 7.4 Hz, 6H, CH_{Ar}), 7.78 (s, 3H, CH_{Ar}), 7.63 (d, J = 7.8 Hz, 6H, CH_{Ar}), 7.08-7.00 (m, 12H, CH_{Ar}), 4.40 (m, 6H, CH_{COD}), 2.94 (m, 6H, CH_{COD}), 2.57-2.27 (m, 36H, CH_3), 2.04 (m, 42H, CH_3, CH_{COD}). ^{13}C{^{1}H} NMR (75 MHz, $CDCl_3$): δ (ppm) 172.1 (Ir- $C_{carbene}$), 146.3 (C_{Ar}), 141.6 (C_{Ar}), 141.3 (C_{Ar}), 141.2 (C_{Ar}), 140.0 (C_{Ar}), 136.5 (C_{Ar}), 135.1 (C_{Ar}), 134.2 (C_{Ar}), 134.0 (C_{Ar}), 132.3 (C_{Ar}), 130.1 (C_{Ar}), 130.0(C_{Ar}), 129.7 (C_{Ar}), 128.3 (C_{Ar}), 127.5 (C_{Ar}), 126.7 (C_{Ar}), 125.3 (C_{Ar}), 81.8 (CH_{COD}), 81.1 (CH_{COD}), 52.8 (CH_{COD}), 50.7 (CH_{COD}), 34.2 (CH_{2COD}), 32.9 (CH_{2COD}), 30.1 (CH_{2COD}), 29.8 (CH_{2COD}), 29.1 (CH_{2COD}), 21.4 (CH_3), 19.6 (CH_3), 18.1 (CH_3), 17.7 (CH_3). HRMS ESI-TOF-MS (positive mode): 1075.6 $[M-Cl]^{2+}$. Anal. Calcd. for $C_{108}H_{117}N_9Ir_3Cl_3(CH_3CH_2CH_2CH_2CH_2CH_3)_3$: C, 60.94; H, 6.46; N, 5.08. Found: C, 60.5; H, 6.8; N, 4.0.

Synthesis of complex 4.
A Schlenk tube was charged with **1** (0.10 g, 0.06 mmol), $NiCp_2$ (0.46 g, 0.24 mmol) and NEt_4Cl (0.30 g, 0.18 mmol). The resulting mixture was suspended in 1,4-dioxane (8 mL) and stirred for 10 min at room temperature. Then, the reaction mixture was heated to reflux overnight and the solution slowly turned from green to dark red. After cooling to room temperature and filtration through celite under nitrogen the solvent was removed under reduced pressure. The obtained solid was washed with diethyl ether and the desired complex was purified by precipitation from a mixture of CH_2Cl_2/hexane, to yield 40 mg of a red solid. Yield: 39.5%. ^{1}H NMR (400 MHz, $CDCl_3$): δ (ppm) 8.44 (bs, 6H, CH_{Ar}), 7.92 (bs, 3H, CH_{Ar}), 7.81 (bs, 9H, CH_{Ar}), 7.18 (bs, 6H, CH_{Ar}), 7.04 (bs, 6H, CH_{Ar}), 4.79 (bs, 15H, CH_{Cp}), 2.36 (bs, 9H, CH_3), 2.23 (bs, 27H, CH_3), 1.99 (bs, 18H, CH_3). ^{13}C{^{1}H} NMR (100 MHz, $CDCl_3$): δ (ppm) 151.4 (Ni-$C_{carbene}$), 148.5 (C_{Ar}), 141.6 (C_{Ar}), 141.4 (C_{Ar}), 140.5 (C_{Ar}), 137.0 (C_{Ar}), 135.5 (C_{Ar}), 134.5 (C_{Ar}), 134.1 (C_{Ar}), 132.0 (C_{Ar}), 130.3 (C_{Ar}), 129.8 (C_{Ar}), 129.4 (C_{Ar}), 127.5 (C_{Ar}), 127.1 (C_{Ar}), 125.6 (C_{Ar}), 125.3 (C_{Ar}), 92.0 (CH_{Cp}), 21.4 (CH_3), 18.3 (CH_3), 17.7 (CH_3). HRMS ESI-TOF-MS (positive mode): 810.7 $[M-2Cl]^{2+}$. Anal. Calcd. for $C_{99}H_{96}N_9Ni_3Cl_3(C_4H_{10}O)_3$: C, 60.56; H, 6.63; N, 6.58. Found: C, 69.5; H, 7.0; N, 6.4.

Synthesis of complex 5.
Into a solution of **3** (0.050 g, 0.02 mmol) in dichloromethane (10 mL) in round a bottomed flask, CO gas was bubbled at 0 °C for 30 min. Then the solution was concentrated under reduced pressure and the addition of hexanes afforded 0.043g of a yellow precipitate. Yield: 92.5%. ^{1}H NMR (300 MHz, $CDCl_3$): δ (ppm) 7.80-7.60 (m, 15H, CH_{Ar}), 7.07-7.04 (m, 12H, CH_{Ar}), 2.41-2.06 (m, 56H, CH_3). ^{13}C{^{1}H} NMR (75 MHz, $CDCl_3$): δ (ppm) 181.0 (Ir-$C_{carbene}$), 168.7 (Ir-CO), 166.0 (Ir-CO), 148.6 (C_{Ar}), 141.8 (C_{Ar}), 141.2 (C_{Ar}), 140.7 (C_{Ar}), 135.8 (C_{Ar}), 134.5 (C_{Ar}), 131.4 (C_{Ar}), 130.3 (C_{Ar}),

129.8 (C_{Ar}), 129.3 (C_{Ar}), 127.0 (C_{Ar}), 125.8 (C_{Ar}), 125.4 (C_{Ar}), 21.3 (CH_3), 18.2 (CH_3), 17.5 (CH_3). HRMS ESI-TOF-MS (positive mode): 2073.5 $[M\text{-}Cl+MeCN]^+$, IR (cm^{-1}): 2057, 1972.

General proceduce for the addition reaction of arylboronic acids to cyclohex-2-enone.

In a 50 mL high-pressure Schlenk tube, catalyst (0.066 mol %), 2-cyclohexen-1-one (0.5 mmol), arylboronic acid (0.6 mmol), KOH (0.09 mmol) and dry toluene (2 mL) were added. The mixture was stirred and heated at 100 °C for 6 h. The reaction yields were calculated by GC, using anisole as internal standard. The identity of the products was confirmed by comparing their ^1H NMR spectra with the ones reported in the literature [2].

References

[1] C. Mejuto, G. Guisado-Barrios, D. Gusev and E. Peris, *Chem. Commun.* **2015**, *51*, 13914-13917.
[2] I. Bratko, G. Guisado-Barrios, I. Favier, S. Mallet-Ladeira, E. Teuma, E. Peris and M. Gómez, *Eur. J. Org. Chem.* **2014**, 2160-2167.

Spectra

^1H and ^{13}C NMR spectra of 2

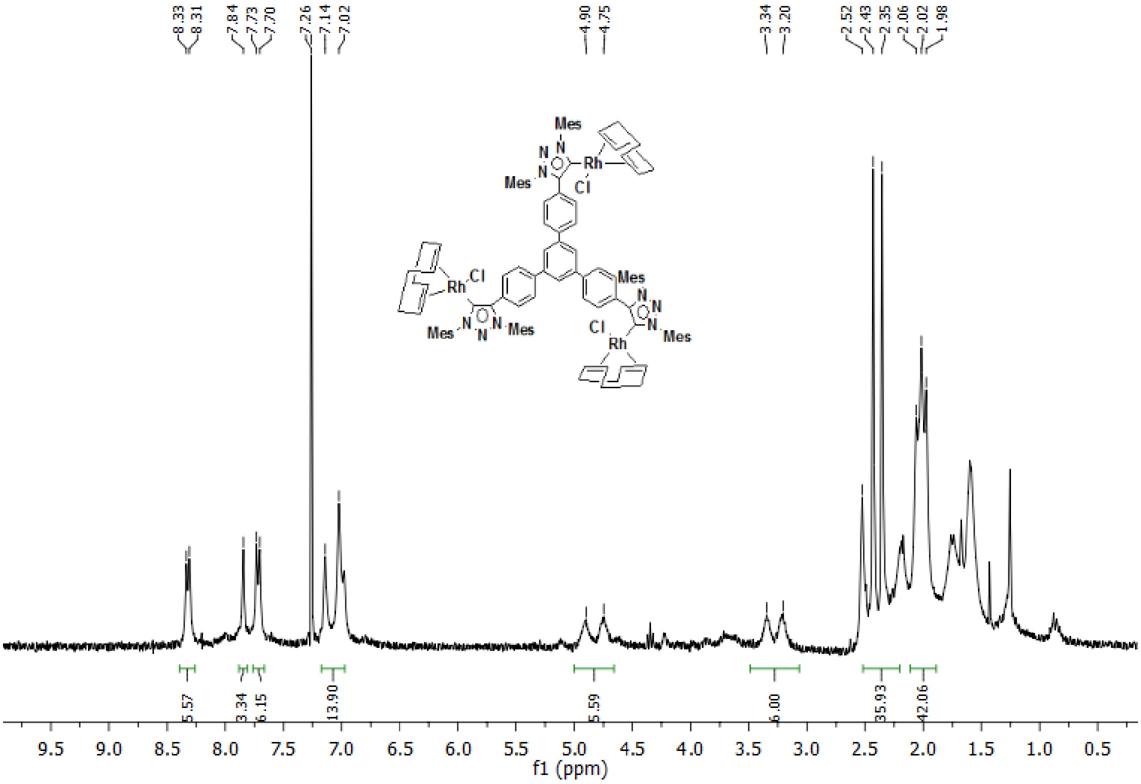

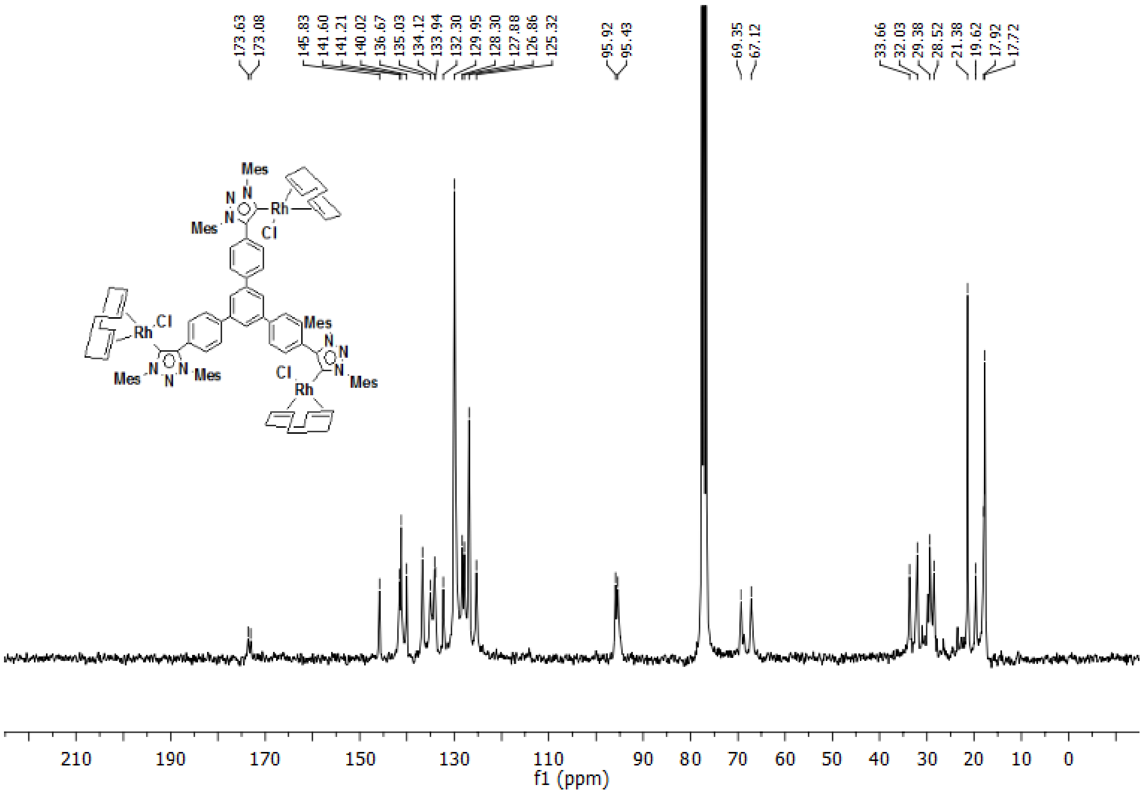

^{1}H and ^{13}C NMR spectra of 3

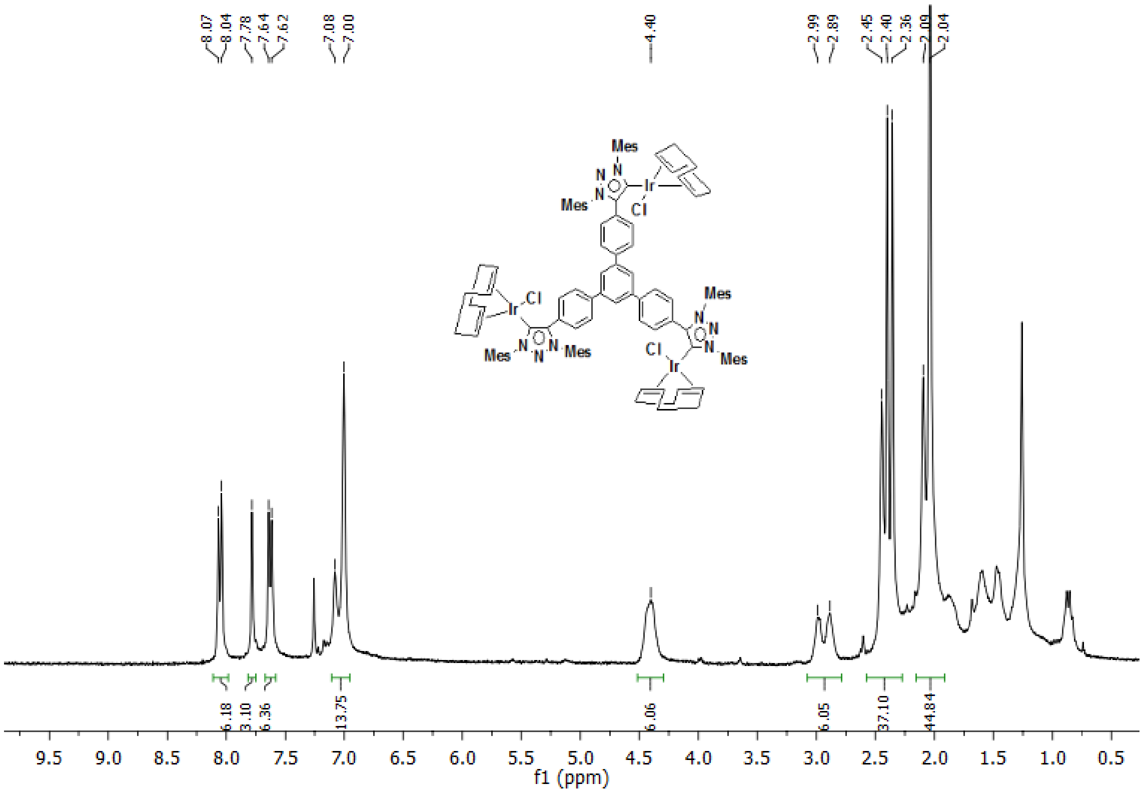

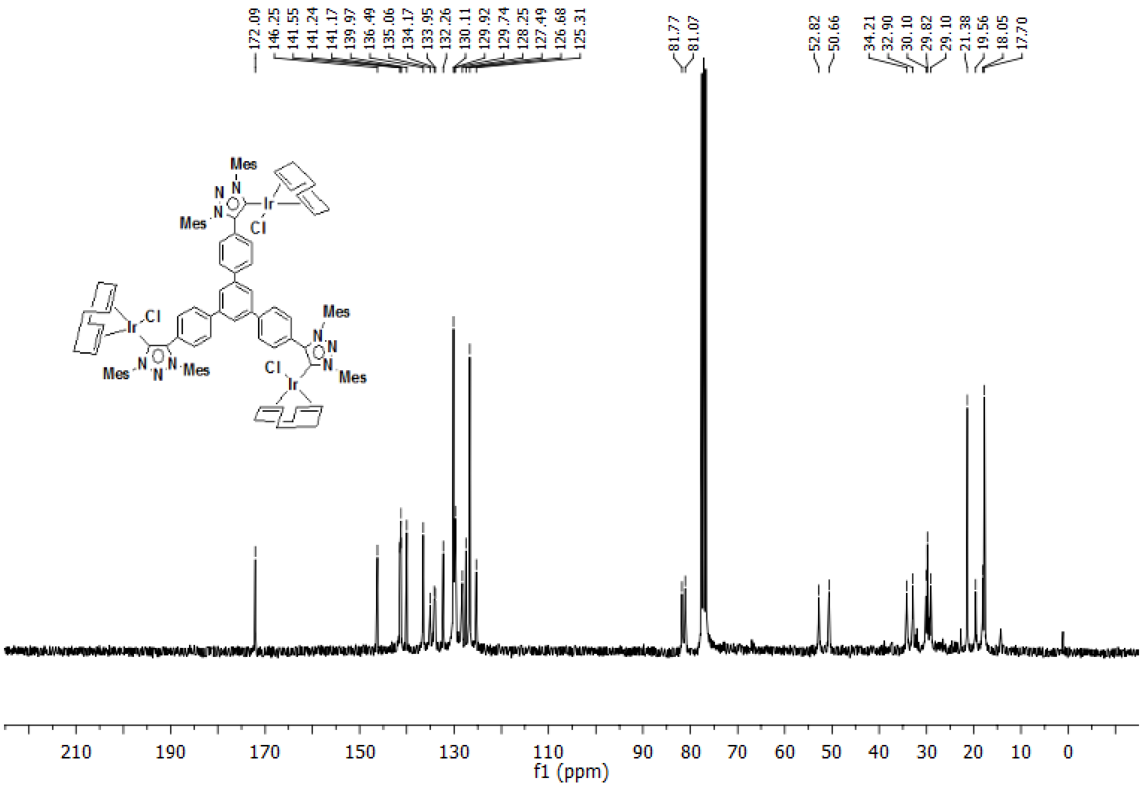

^{1}H and ^{13}C NMR spectra of 4

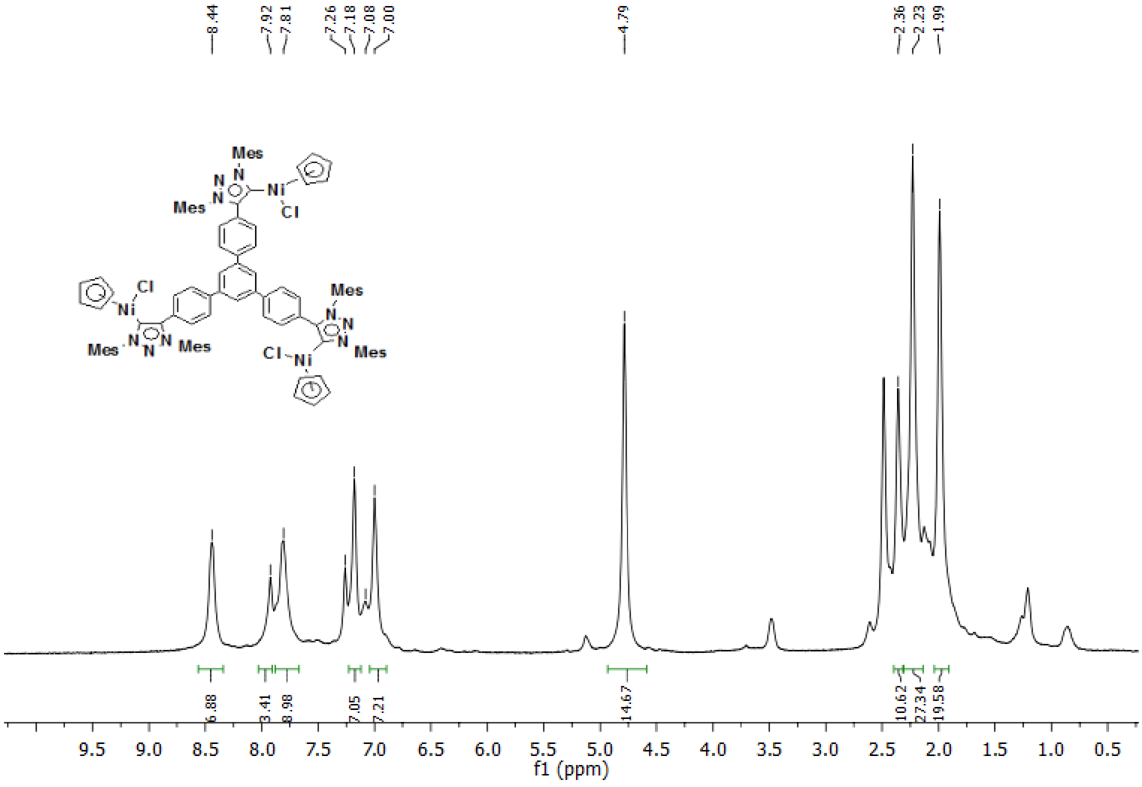

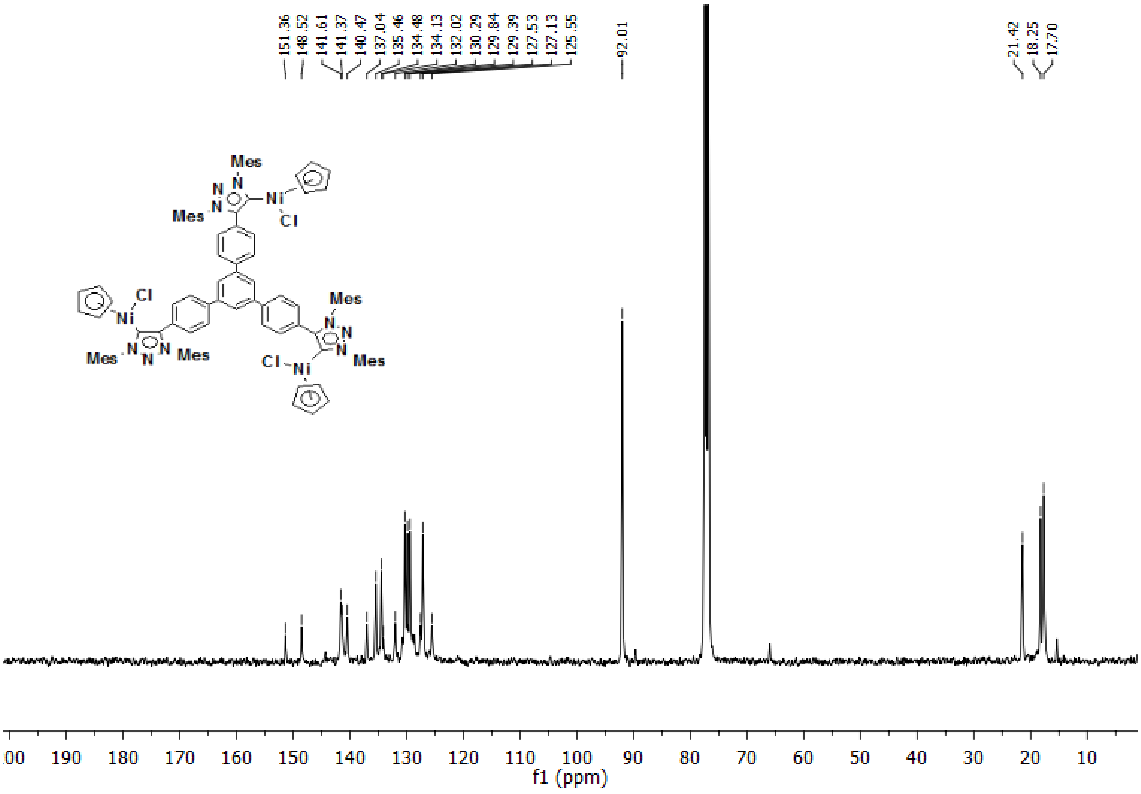

^{1}H and ^{13}C NMR spectra of 5

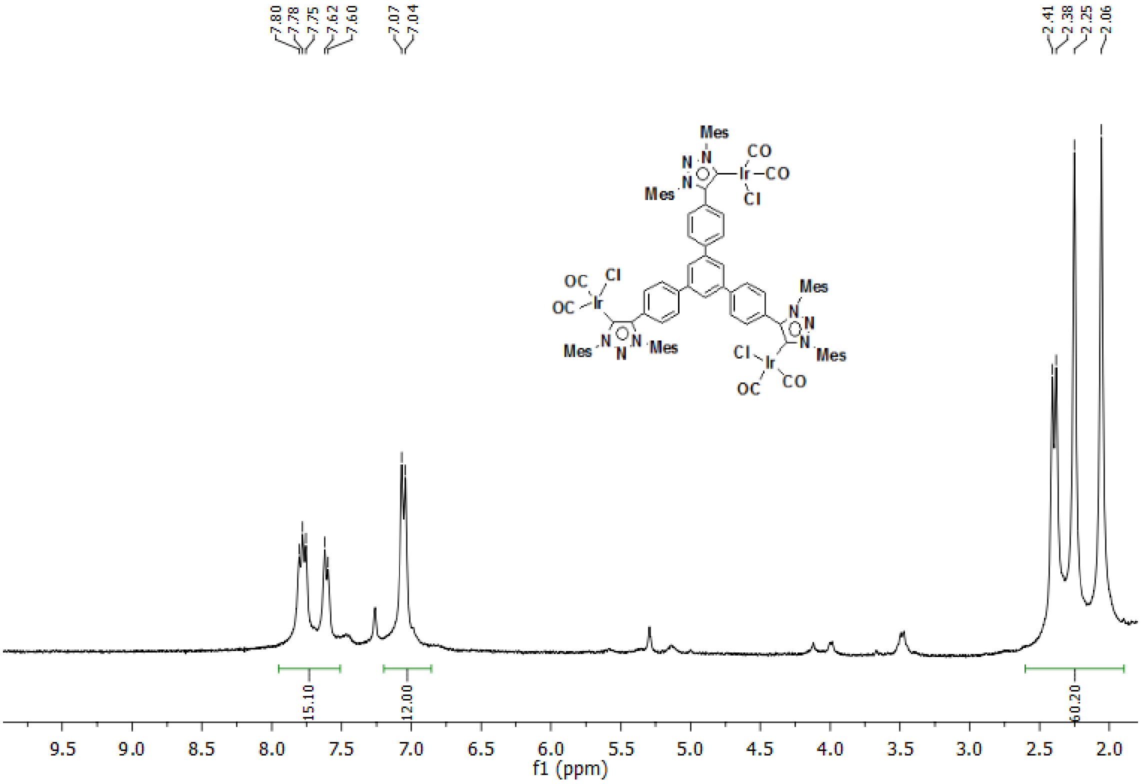

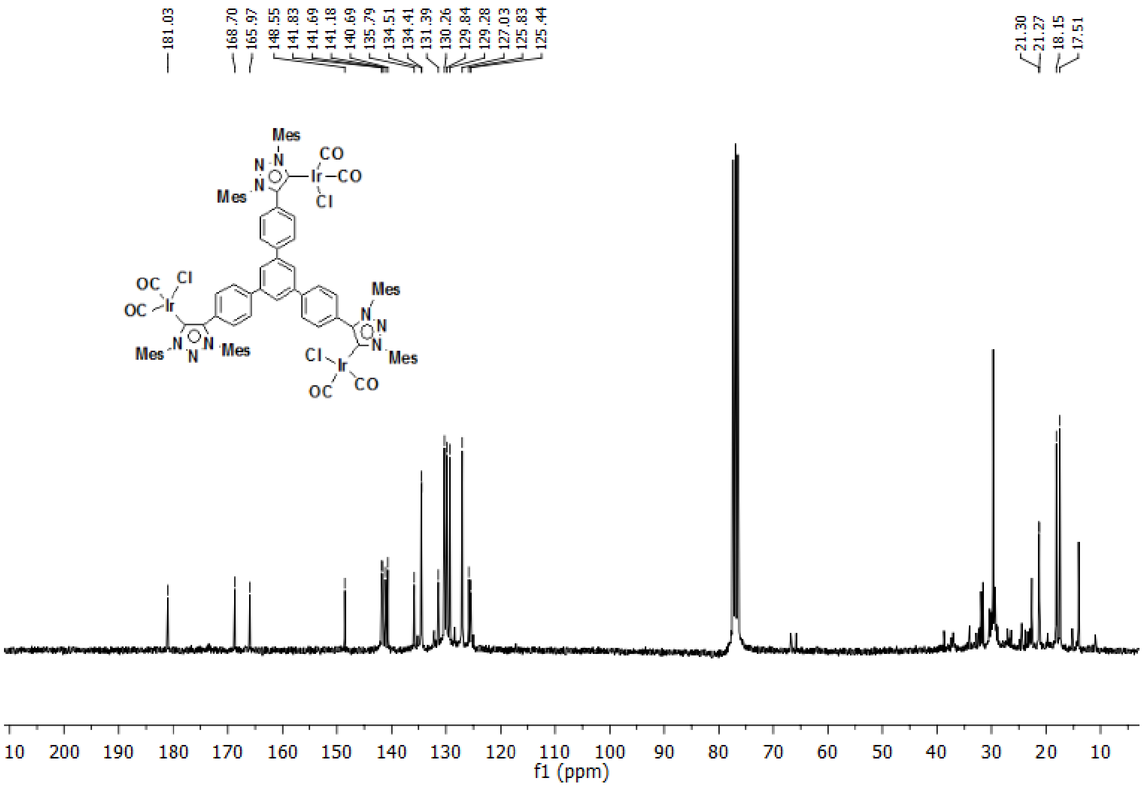
181.03
168.70
165.97
148.55
141.83
141.69
141.18
140.69
135.79
134.51
134.41
131.39
130.26
129.84
129.28
127.03
125.83
125.44
21.30
21.27
18.15
17.51
Mes
N
CO
Ir
Cl
OC
10 200 190 180 170 160 150 140 130 120 110 100 90 80 70 60 50 40 30 20 10
f1 (ppm)
